# Comprehensive school-based health programs to improve child and adolescent health: Evidence from Zambia

**DOI:** 10.1371/journal.pone.0217893

**Published:** 2019-05-31

**Authors:** Dorothy Wei, Rachel Brigell, Aayush Khadka, Nicole Perales, Günther Fink

**Affiliations:** 1 Department of Global Health and Population, Harvard T. H. Chan School of Public Health, Boston, Massachusetts, United States of America; 2 Department of Health Policy, University of California Berkeley School of Public Health, Berkeley, California, United States of America; 3 Deparment of Epidemiology and Public Health, Swiss Tropical and Public Health Institute, University of Basel, Basel, Switzerland; St Francis Hospital, UNITED STATES

## Abstract

**Background:**

While school-aged children in low- and middle-income countries remain highly exposed to acute infections, programs targeting this age group remain limited in scale and scope. In this paper, we evaluate the impact of a new and comprehensive primary school-based health intervention program on student-reported morbidity and anthropometric outcomes in Lusaka, Zambia.

**Methods:**

A prospective matched control study identified 12 classes in 7 schools for the intervention and 12 classes in 7 matched schools as controls. Teachers in intervention schools were trained to deliver health lessons and to refer sick students to care. In addition, vitamin A and deworming medication were biannually administered to intervention students. The primary study outcome was student-reported morbidity. Secondary outcomes were weight, height, health knowledge, and absenteeism. Multivariable linear and logistic regression models were used to estimate program impact.

**Results:**

380 students ages 4–16 were enrolled in the study in 2015, and 97% were followed up at endline in 2016. The intervention decreased the adjusted odds of self-reported acute illnesses by 38% (95% CI: 0.48, 0.77) and the adjusted odds of stunting by 52% (95% CI: 0.26, 0.87). It also increased health knowledge by 0.53 standard deviations (95% CI: 0.24, 0.81). No impact was found on weight (adjusted mean difference β = 0.17, 95% CI: - 1.11, 1.44) and student absenteeism (adjusted odds ratio (aOR) = 0.89, 95% CI: 0.60, 1.33).

**Conclusion:**

The results presented in this paper suggest that comprehensive school-based health programs may offer a highly effective way to improve students’ health knowledge as well as their health status. Given their low cost, a more general adoption and implementation of such programs seems recommendable.

**Trial registration:**

ClinicalTrials.gov Identifier: NCT03607084.

## Introduction

While child health has been a key global health priority in the past two decades, global child health efforts have largely focused on the first five years of life, when children face the highest mortality risk [1–4]. However, as efforts to reduce child mortality continue with the Sustainable Development Goals, the need to address child and adolescent health more broadly has been increasingly recognized [4–6]. One area of particular importance is late childhood and early adolescence, which is a time of significant emotional, social, intellectual, and physical development, and a period where health adversity has been shown to result in increased school absenteeism, diminished academic performance, impeded intellectual and physical growth, and slowed skills-development [1,3,7,8].

Although evidence on the average health status of school-aged children in low- and middle-income countries (LMIC) is very limited, exposure to malnutrition and infectious diseases remains high in many settings [2,9–12]. Globally, an estimated 20–30% of school-aged children in LMICs are stunted and over 600 million school-aged children live in areas endemic of parasitic worms and are need of treatment [1,13].

Whereas routine child health checkups have been developed and implemented for children under the age of five in many countries, standards of care for older children are less defined [12]. The World Health Organization, the World Bank, and United Nations Educational, Scientific and Cultural Organization have promoted schools as a platform to deliver interventions to children and adolescents; due to the rapid increase in primary school enrollment attained in the past two decades, the potential reach of this platform is large [7,14,15].

While basic school health programs are implemented almost universally, many national school health programs suffer from poor funding, low coverage, and irregular implementation [16–20]. In this study, we assess the extent to which comprehensive primary school-based health programs can improve child and adolescent health in LMIC settings.

## Materials and methods

The study protocol for this trial and supporting TREND checklist are available as supporting information; see S1 Checklist and S1 Protocol. The trial is registered at ClinicalTrials.gov (NCT03607084).

### Study setting

The study was conducted in primary schools located in the Ng’ombe, Chaisa, Garden, and Mandevu neighborhoods (“compounds”) in the peri-urban outskirts of Lusaka, Zambia between 2015 and 2016. These compounds, or informal settlements, are characterized by high poverty, poor sanitation, over-crowding, and inadequate infrastructure and access to basic services [21,22].

In terms of the larger national context, Zambia is fairly representative of LMICs in the African region. Zambia ranks at the 10^th^ percentile globally with respect to primary school net enrollment rate and youth literacy rate [23]. 64% of its youth are literate, which falls below the average youth literacy rate across all LMICs [23]. While primary school enrollment and attendance is high in Zambia, 39% of Zambian youth fail to complete primary education [23]. Helminth infections, diarrheal diseases, respiratory illnesses, and malaria are highly prevalent among Zambian children over the age of five [24]. With 50% of the Zambian population under the age of 20, the predominately young population poses additional challenges to the overstrained health and education sectors [25].

### Intervention

To address the gap in care for school-aged children in Zambia, a comprehensive school-based health intervention was developed by a local non-governmental organization (NGO) with support of the government in 2014. The school-based health program has two main components: a School Health Worker (SHW) program and bi-annual health screenings. The SHW program trains selected teachers to deliver health lessons to students, perform basic first aid, recognize common illnesses among students, identify warnings signs and refer these cases to skilled medical attention. The student health lessons cover nutrition, sanitation, malaria, tuberculosis, and HIV/AIDS. SHWs are also trained to improve school water, sanitation, and hygiene conditions, distribute oral rehydration solution, and provide instruction on its use. Selected teachers complete an initial 100 hours training course and are then mentored and monitored on a continued basis by the non-governmental organization. All schools with trained SHWs are provided with a 100 Zambian kwacha monthly allowance (~$17), basic medical supplies including pain relief medication, thermometers, bandages, antiseptics, and oral rehydration solution.

In addition to the SHW activities, school-wide health screenings are implemented every six months at schools with support from nurses and clinical officers from a local clinic. As part of these health screenings, all students are provided with vitamin A supplementation and presumptive deworming medication. Clinic staff also perform anthropometric measurements and evaluate children for signs and symptoms of acute respiratory, fungal, urinary tract, and eye infections, as well as diarrheal diseases, malaria, tuberculosis, and other acute and chronic conditions. Urine screening for schistosomiasis is also performed. Students who test positive for schistosomiasis or present with symptoms of other illnesses are either given medication at the health screening, or referred to the clinic if the illness that cannot be treated at the screening.

### Study design

A prospective matched control study was used to assess program impact on student outcomes. The sampling for the study was done in two steps. In the first step, seven schools from the Ng’ombe compound were selected by the NGO in collaboration with the local ministry of health and school directorate to receive the intervention. In the second step, and prior to the launch of the program, matched control schools were identified by the research team for each of the seven schools. Matching was based on minimum unweighted average standardized distances for matching factors that reflect educational climate and resources. Specifically, for each of the seven intervention schools, a matched control school that was most similar in terms of the schools’ number of teachers, student population, and passing rate, was selected. Infrastructure variables such as water and sanitation were also considered, however we found no meaningful differences across schools to inform the matching process. After the matching process, intervention and control schools were compared to ensure baseline balance: no differences by intervention status were found in either of the three matching variables considered (S1 Table). The study was powered to detect a decline in infection prevalence from an expected baseline level of 0.50 to 0.35, assuming a design effect of two as well as an attrition rate of 10% over the study period. All study schools were located within the Chipata sub-district zone, one of six administrative zones in Lusaka.

### Participants

From each intervention school, two grades directly taught by School Health Workers were randomly selected for the study. The grades in the control schools were selected to match those in the intervention schools. One of the intervention schools had only one SHW, therefore one grade was selected from this school and its paired control school. All students in the selected grades were invited to participate in the study. 50% of students from intervention schools and 51.1% of students from control schools agreed to participate in our study. Prior to data collection, parental consent forms in English and Nyanja were distributed to all classes selected to participate in the study. Students who returned signed consent forms from a parent or guardian were asked to sign an assent form that was read aloud to them in both English and Nyanja. Those who gave assent were enrolled in the study.

### Data collection

Data was collected across two time-points during 2015–2016: baseline (prior to intervention) and endline (one year after program launch) using face-to-face interviewer-administered surveys. Both survey rounds collected anthropometric measurements, self-reported data on health status, and data on school attendance. Trained nurses, clinical officers, and pharmacists from the Ng’ombe Community Health Centre visited the study schools to interview participants. Surveys also covered family characteristics, socioeconomic status, health knowledge, academic performance, healthcare utilization, absenteeism, and presence of acute and chronic diseases. Weight and height were directly assessed by study staff using standard anthropometric measurement kits.

### Outcomes

#### Health outcomes

The primary outcome of this study was self-reported morbidity. As part of the survey, all students were asked to report on 14 systemic, genitourinary, respiratory, and gastrointestinal illnesses, which were combined into a summary index of morbidity (S1 Appendix). Secondary outcomes were weight and height, which the study team measured at baseline and endline, as well as observed health knowledge and self-reported absenteeism. For weight, we analyzed absolute weight, as well as binary indicators for thinness and overweightness. For height, the main outcome measure was stunting. We calculated standardized height-for-age (HAZ) z-scores and body mass index (BMI)-for-age z-scores using the World Health Organization AnthroPlus Software macro for Stata. These z-scores were converted into binary indicators for stunting, thinness, and overweightness, which are defined respectively as: HAZ below -2 standard deviations (SD), BMI-for-age below -2 SD, and as BMI-for-age above 1 SD. To measure general absenteeism, participants were asked whether they had missed one or more days from school in the past two weeks. Health knowledge was assessed with an 11-question quiz on various health topics (S2 Appendix). We calculated the percentage of correct answers on the 11-point score, and then converted this percentage into a z-score to standardize and ease the interpretation of coefficients.

#### Access to health services

Information on receipt of deworming medication and vitamin A supplementation in the six months preceding data collection was directly provided by students. To measure students’ perceptions of teacher support, participating students were asked to describe the level of ease or difficulty in receiving treatment when sick (1- Difficult, 2- Somewhat difficult, and 3- Easy). Participants were also asked whether they agree that teachers can help students with their health when sick. We coded both variables into primary indicators for “easy access” and “teacher can help when sick.”

### Statistical analysis

To assess the impact of the intervention on health and schooling outcomes, we used logistic regression models for binary outcomes and Ordinary Least Squares (OLS) regression models for continuous outcomes. For the self-reported morbidity outcomes, we restricted the analysis to variables with at least 5% prevalence at baseline and endline. To analyze the 11 self-reported morbidity outcomes, we first estimated illness-specific logistic models and then used random effects meta-analysis to aggregate the results and estimate overall (average) program impact on morbidity. For all outcome variables, both adjusted and unadjusted models were estimated. Adjusted models included controls for age (years), gender, grade, family wealth quintile, and all access to health services indicators and health outcomes at baseline. Since analyses were performed at the individual-level, standard errors for all regressions were clustered at the school level using Huber’s cluster robust variance estimator to account for the clustered sampling design used in the evaluation [26]. Missing data was imputed for all baseline controls (16 variables) using multiple imputation. Multivariate normal imputation was implemented using 250 imputations. The analysis was restricted to all non-graduating students (students below grade 7) and focused on the health outcomes as described in the study protocol (S1 Protocol). All analyses were performed using Stata Version 14 [27].

## Ethical considerations

The study was approved by the Institutional Review Board of the Harvard T.H. Chan School of Public Health in Boston, Massachusetts and the Excellence in Research Ethics and Science (ERES) Converge Research Ethics Committee, Ministry of Education, and District Education Board Secretary in Lusaka, Zambia.

## Results

**Fig 1:**
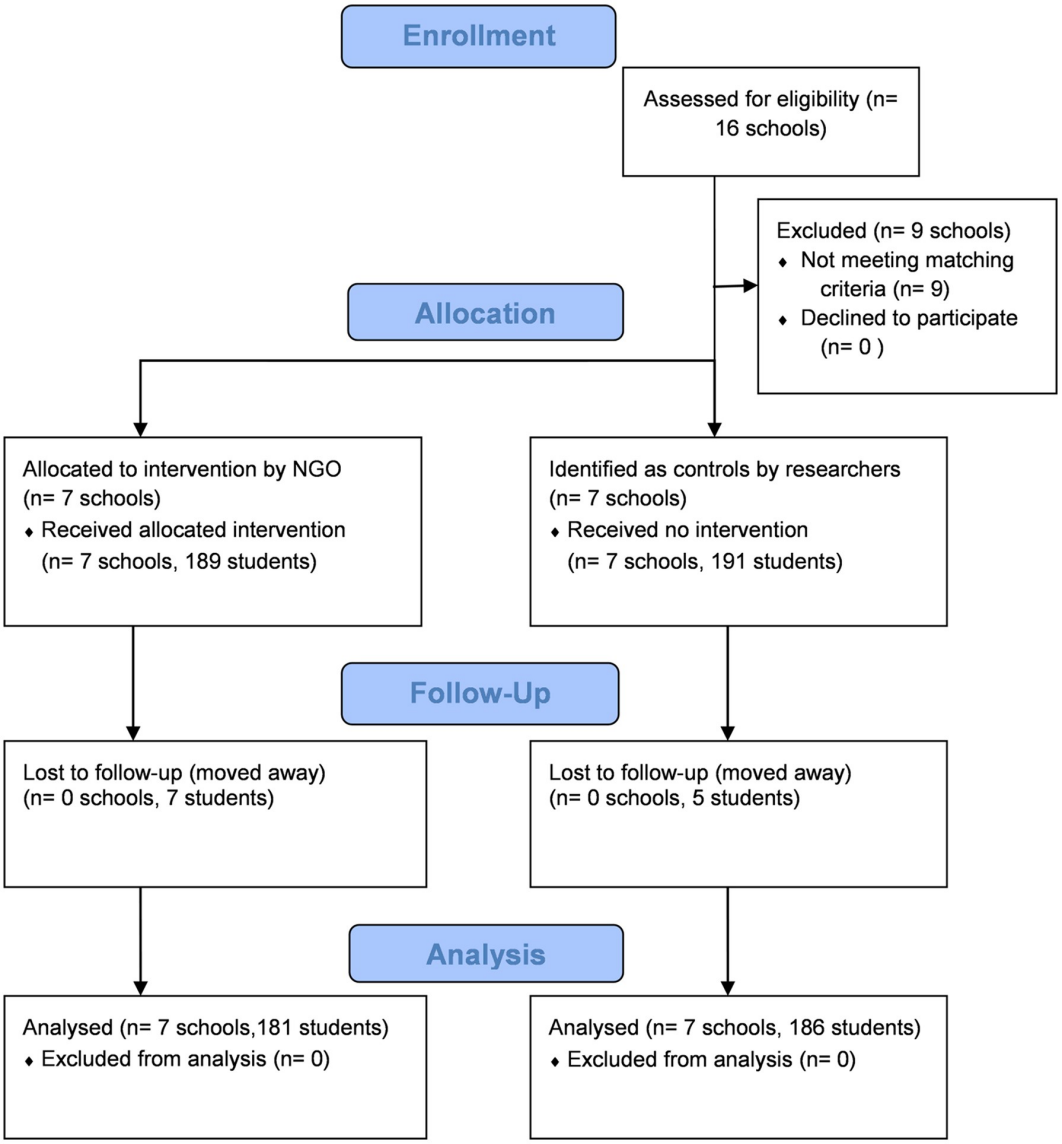
Sampling procedure of school-aged children in Lusaka, Zambia, 2015–2016.

380 students were enrolled into the study between July and August 2015. 97% of study participants (N = 367) were followed-up at one year after program launch during July and August 2016. Fig 1 provides an overview of the sample sizes by intervention and control groups across survey rounds.

Baseline characteristics of students were similar in the intervention and control groups (Table 1). In the full sample, 52% of the students were female and 42% were above grade 3 (grades 4–6), with an average age of 9.77 years and from households with a mean wealth quintile of 2.94. Intervention students were marginally older than control students at baseline (p-value = 0.39). At baseline, 37% of students reported to have received deworming medication and 38% received vitamin A supplementation in the past six months. 15% of students were stunted, 13.5% were overweight, and 4% were thin. Nearly 94% of students agreed that teachers can help students when sick at baseline, and 78% of students perceived their access to care as easy. About 48% of students reported missing one or more days from school due to unspecified reasons two weeks prior to the survey.

**Table 1:** Baseline characteristics of study participants.

Characteristic	Full Sample (N = 380)			Control (N = 191)		Intervention (N = 189)		P-value*
	N	Mean (SD)	%	Mean (SD)	%	Mean (SD)	%	
*Demographics*								
Female	380		52.11		51.85		52.36	0.76
Age (years)	380	9.77 (2.56)		9.32 (2.44)		10.2 (2.62)		0.39
Above Grade 3	380		42.36		39.67		46.67	0.66
Wealth quintile	370	2.94 (1.4)		2.84 (1.4)		3.04 (1.39)		0.43
*Access to Health Services*								
Received deworming medication in past 6 months	375		37.07		32.98		41.18	0.45
Received vitamins at school in past 6 months	373		38.07		35.71		42.55	0.27
Confidence in teacher’s ability to help with health	373		93.83		92.97		94.68	0.94
Perceived access to care as easy	373		78.02		81.7		74.33	0.45
*Outcomes*								
Weight (kg)	377	29.1 (8.63)		28.6 (8.72)		29.79 (8.52)		0.62
Thin	378		3.97		2.67		5.23	0.80
Overweight	378		13.49		17.11		9.95	0.38
Stunted	378		15.08		11.80		18.3	0.38
Absent	378		47.62		47.06		48.17	0.76
Health knowledge (z-score)	380	0 (1)		-0.17 (1.04)		0.16 (0.93)		0.099

* p-value based on a two-sample mean comparison test with standard errors clustered at the school level.

### Health outcomes

**Fig 2:**
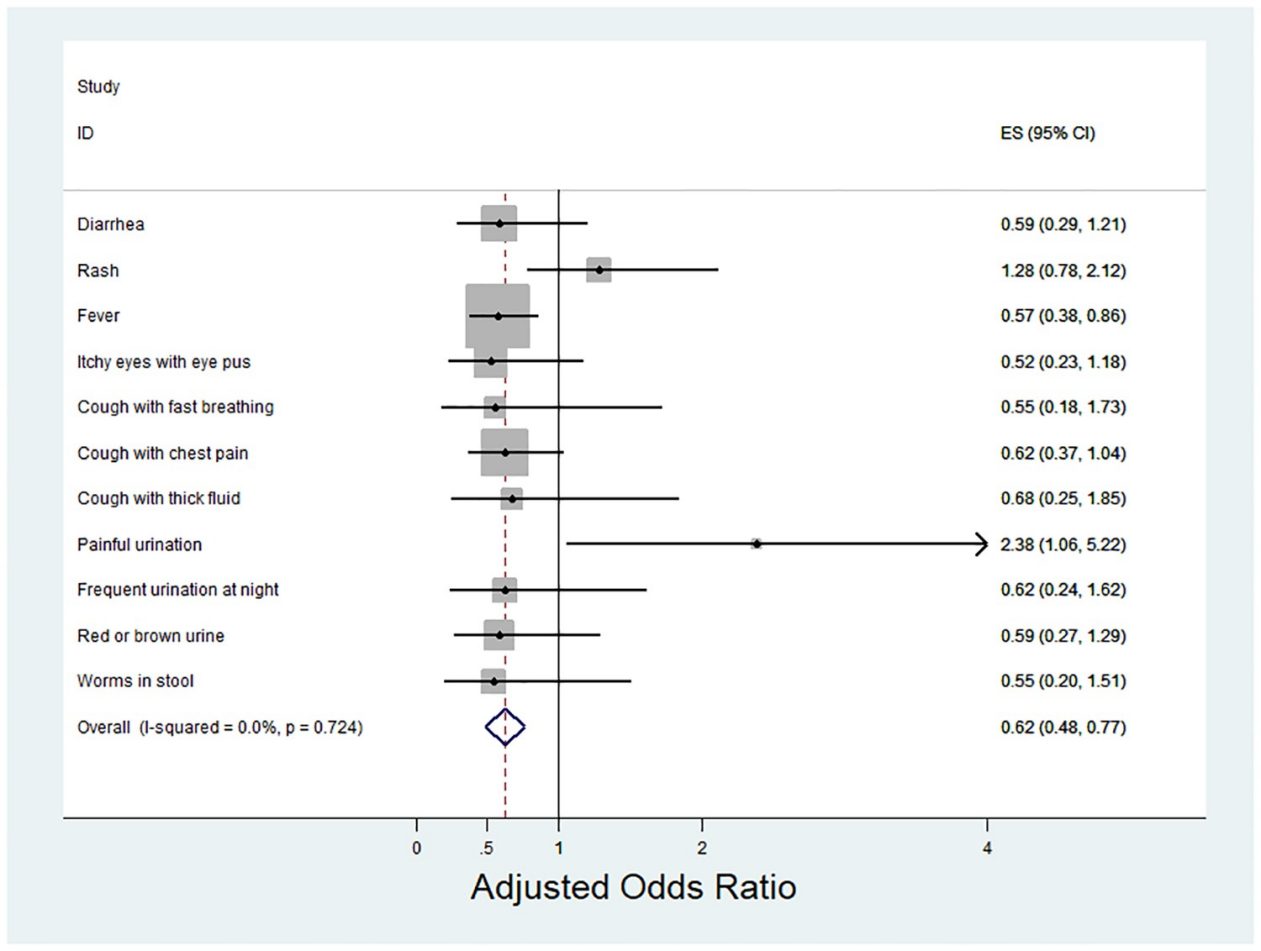
Meta-analysis of estimated morbidity reductions. Meta-analysis of estimated morbidity reductions among school-aged children in Lusaka, Zambia, 2015–2016. The overall estimate is based on random-effect meta-analysis. The coefficients and confidence intervals underlying this meta-analysis were computed through separate logistic regression models for each morbidity measure. All logistic models controlled for the illness outcome at baseline and baseline values of: age, grade, sex, wealth quintiles, deworming, vitamin A, confidence in teachers, perceived access to care, weight, thinness, overweight, stunting, health knowledge score, and absenteeism.

Fig 2 presents a forest plot of adjusted odds ratios for the program impact on acute illnesses at one-year follow-up. Each line in the forest plot represents the estimated program impact on one of the 11 morbidity outcomes. The overall program impact on morbidity, estimated through the random effects meta-analysis of the 11 illness-specific logistic regressions, suggest that on average, the program lowered the adjusted odds of a student reporting health problems by 38% (adjusted odds ratio (aOR) = 0.62, 95% CI: 0.48, 0.77). Notably, the intervention lowered the adjusted odds of fever by 42.8% (aOR = 0.57, 95% CI: 0.38, 0.86) and the adjusted odds of cough with chest pain by 38% (aOR = 0.62, 95% CI: 0.37,1.04). Changes in the absolute prevalence of illness outcomes by intervention status are detailed in S2 Table.

**Fig 3:**
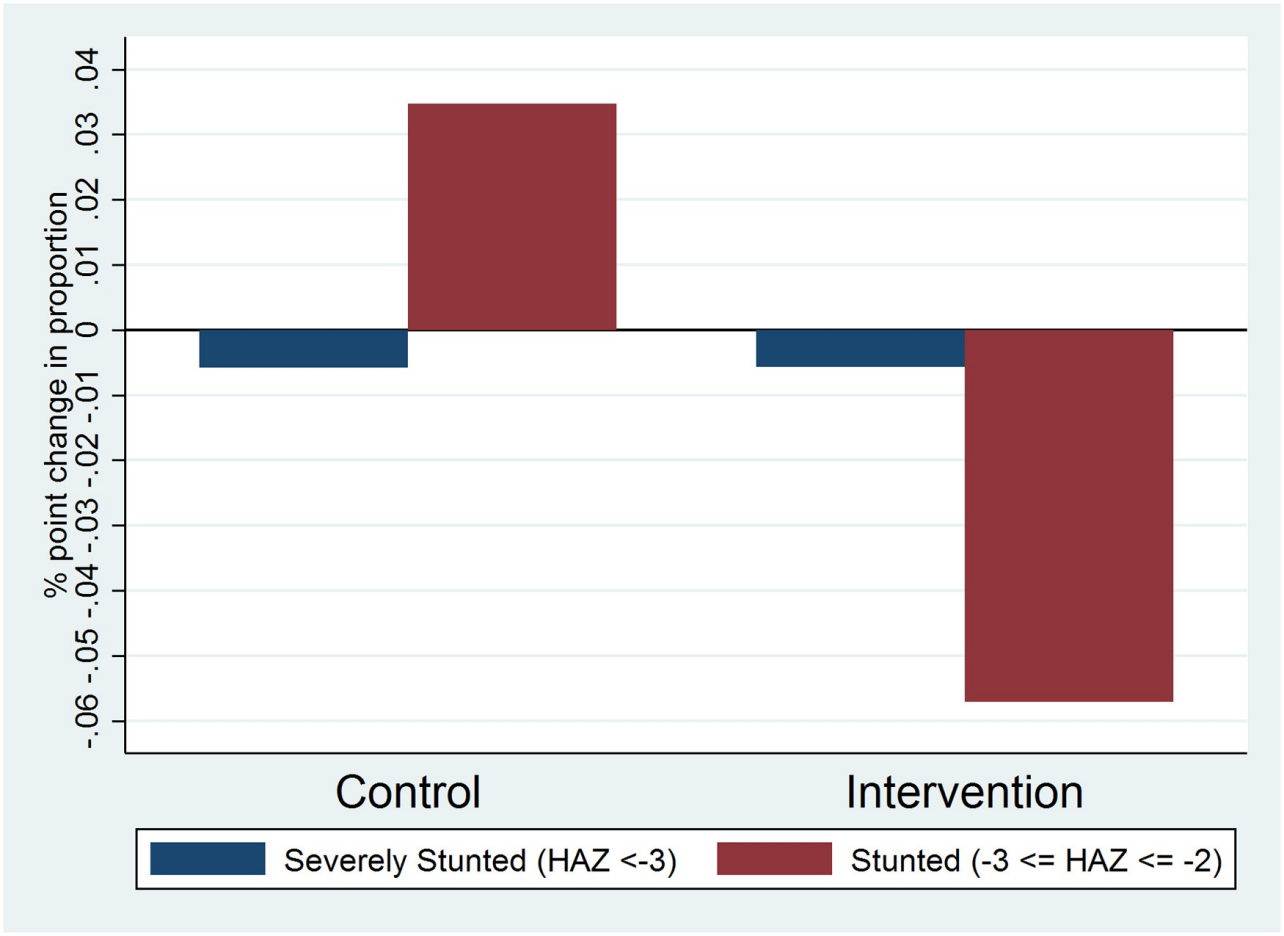
Change in proportion of children in stunting category from baseline to endline. Changes in the prevalence of stunting and severe stunting between baseline and endline for intervention and control groups in Lusaka, Zambia, 2015–2016.

Table 2 shows estimated results for anthropometric outcomes. No impact was found on weight (adjusted mean difference (β) = 0.17, 95% CI: - 1.11, 1.44), overweight (aOR = 0.54, 95% CI 0.23, 1.30), and thinness (aOR = 1.03, 95% CI: 0.27, 3.94). However, the intervention decreased the adjusted odds of being stunted by 52% (aOR = 0.48, 95% CI: 0.26, 0.87). Fig 3 shows the change in stunting trends across two HAZ z-score groups by intervention status. Among intervention students, severe stunting (HAZ <-3) decreased by 0.57 percentage points and non-severe stunting (-3 ≤ HAZ ≤ -2) decreased by 5.71 percentage points from baseline to endline. In the control group, a similar decrease in severe stunting was observed, however the proportion of non-severely stunted students increased by 3.5 percentage points over time.

**Table 2:** Intervention impact on anthropometric outcomes.

	Unadjusted Model	Adjusted Model
*Weight (kg)*		
Mean difference	1.41	0.17
95% CI	(-6.03–8.85)	(-1.11–1.44)
Mean among controls	31.60	31.60
N	361	361
*Thin*, *BMIZ < -2 (%)*		
OR	1.40	1.03
95% CI	(0.56–3.51)	(0.27–3.94)
% among controls	4.57%	4.57%
N	350	350
*Overweight*, *BMIZ > 1 (%)*		
OR	0.33**	0.54
95% CI	(0.13–0.86)	(0.23–1.30)
% among controls	12.00%	12.00%
N	350	350
*Stunted*, *HAZ < -2 (%)*		
OR	0.86	0.48***
95% CI	(0.41–1.81)	(0.26–0.87)
% among controls	14.29%	14.29%
N	350	350

*** p<0.01,

** p<0.05,

* p<0.1

Note: Estimates from linear (weight) and logistic (all other outcomes) regression. Adjusted models controlled for baseline values of: age, grade, sex, wealth quintiles, deworming, vitamin A, confidence in teachers, perceived access to care, weight, thinness, overweight, stunting, health knowledge score, and absenteeism. 95% confidence intervals shown in parentheses were clustered at the school level using Huber’s cluster robust variance estimator.

Table 3 shows the program impact on health knowledge and absenteeism. The intervention improved health knowledge by a 0.53 SD increase in knowledge score (95% CI: 0.24, 0.81). No relationship was found between the intervention and general absenteeism (aOR = 0.89, 95% CI: 0.60, 1.33).

**Table 3:** Intervention impact on health knowledge and absenteeism.

	Unadjusted Model	Adjusted Model
*Health knowledge (z-score)*		
Mean difference	0.62**	0.53***
95% CI	(0.06–1.18)	(0.24–0.81)
Mean among controls	-0.31	-0.31
N	364	364
*Absent (%)*		
OR	0.96	0.89
95% CI	(0.62–1.50)	(0.60–1.33)
% among controls	43.72%	43.72%
N	363	363

*** p<0.01,

** p<0.05,

* p<0.1

Note: Estimates from linear (health knowledge) and logistic (absent) regression.

Adjusted models controlled for baseline values of: age, grade, sex, wealth quintiles, deworming, vitamin A, confidence in teachers, perceived access to care, weight, thinness, overweight, stunting, health knowledge score, and absenteeism. 95% confidence intervals shown in parentheses were clustered at the school level using Huber’s cluster robust variance estimator.

### Access to health services

Table 4 displays results from the treatment coverage and teacher support regression models. The intervention increased the proportion of students receiving deworming by 48 percentage points (aOR = 11.24, 95% CI: 4.84, 26.15) and the proportion of students receiving vitamin A supplementation by 47 percentage points (aOR = 11.19, 95% CI: 5.18, 24.17). The intervention also increased the adjusted odds of students being confident in their teacher’s ability to help with health problems by a factor of 4.49 (95% CI: 1.77, 11.38) and the adjusted odds of perceiving access to care as easy by 2.99-fold (95% CI: 1.42, 6.31). Changes in deworming and vitamin A coverage over time are detailed in S1 Fig. While the proportion of students not receiving vitamin A in the past six months decreased from 57% to 14% over the study period in the intervention group, no improvements were seen in the control group. Similarly, the proportion of students not receiving deworming medication decreased from 59% to 16% in the intervention group, whereas it remained above 63% in the control group at endline.

**Table 4:** Intervention impact on access to health services.

	Unadjusted Model	Adjusted Model
*Deworming*		
OR	9.24***	11.24***
95% CI	(4.43–19.27)	(4.84–26.15)
% among controls	37.10%	37.10%
N	352	352
*Vitamin A*		
OR	9.50***	11.19***
95% CI	(5.0–18.03)	(5.18–24.17)
% among controls	37.64%	37.64%
N	353	353
*Confidence in teacher’s ability to help with health*		
OR	3.85***	4.49***
95% CI	(1.58–9.37)	(1.77–11.38)
% among controls	83.15%	83.15%
N	358	358
*Perceived easy access to care*		
OR	2.51***	2.99***
95% CI	(1.30–4.86)	(1.42–6.31)
% among controls	80.11%	80.11%
N	354	354

*** p<0.01,

** p<0.05,

* p<0.1

Note: Estimates from logistic regression. Adjusted models controlled for baseline values of: age, grade, sex, wealth quintiles, deworming, vitamin A, confidence in teachers, perceived access to care, weight, thinness, overweight, stunting, health knowledge score, and absenteeism. 95% confidence intervals shown in parentheses were clustered at the school level using Huber’s cluster robust variance estimator.

## Discussion

The results from this study have yielded four main results: First, as documented in other studies, the prevalence of infections is substantial among primary school students [1,2,7–10,28]. At baseline, 23% of students reported experiencing diarrhea, 24% reported the presence of worms in their stool, 35% reported having a fever, and 42% of students reported having a cough with chest pain in the two weeks preceding the baseline survey. Second, despite national efforts to provide vitamin A supplementation and deworming for all children, access to these health services appears to be limited among school-aged children in the studied setting, with only 37% of students reporting receipt of deworming and 38% reporting vitamin A supplementation in the past six months at baseline.

Third, and more positively, our analysis suggest that relatively simple school-based health programs can lead to substantial improvements in children’s health. The results presented here suggest that the program reduced overall average morbidity by 38% and lowered stunting prevalence by 52%. Although the impact on stunting appears large, it is important to note that the majority of children in this sample are rather close to the -2 SD HAZ cutoff. 45.2% of students had a HAZ between -1 and -3 at baseline, which suggests that small differences in linear growth can have potentially large impacts on stunting prevalence. Our results also imply that recovery from growth faltering is possible throughout childhood and adolescence as suggested by the more recent literature on physical growth trajectories [2,29–32]. While a recent systematic review and meta-analysis conclude that regular deworming has little to no effect on average height, many of the included studies had either no or limited co-interventions [33,34]. The program analyzed in this study, however, is a comprehensive intervention package of health screenings, preventive treatment, health education, and teacher trainings. It is possible that the SHW program contributed to reductions in stunting due to the improved ability of teachers to identify, refer, and treat cases of acute illnesses that negatively impact nutritional status and growth, such as diarrheal diseases.

Finally, regarding observed morbidity improvements, our findings are consistent with a growing body of evidence demonstrating that small investments in micronutrient supplementation, school feeding programs, and skills-based health education can increase access to care, positively affect health promoting behaviors, improve nutritional status and linear growth, and reduce illnesses [9,15,35–39]. School-based health programs may have a broader impact beyond schools and its students. Teachers often work closely with parents and the community, and students are likely to share health lessons in their homes and communities [15].

Regarding overall resources needed, school-based health programs can be implemented at a low cost. In the case of the program analyzed, the average total cost per child for two health screenings, which includes deworming, vitamin A, and a physical assessment, was $0.70 during the first year of implementation (S3 Table). The inclusion of the SHW program along with the bi-annual health screenings increased the average cost per child to $5.10 during the first year. These estimates include the start-up cost of teacher training and equipment. A unique feature of the program analyzed was the training of teachers to become school health workers, which seems to have resulted in considerable improvements in health knowledge and health access. After initial investments, the recurrent cost decreased to $0.45 per child for health screenings and $2.10 per child for both SHW and screening programs.

This study is not without limitations. First, our study used self-reported measures of morbidity and absenteeism, which may be subject to recall and social desirability bias. These concerns are heightened given the young age of our study population. Our results reveal a high burden of acute illnesses among school-aged children, however it is possible that students may have overstated their morbidity. To minimize recall bias and improve comprehension, our study provided clear and easy to understand definitions and repeated survey questions to respondents. Second, we evaluated the impact of the program in its entirety and therefore are unable to distinguish the individual effects of different program components. While the improvements in health knowledge and perceived ease of access can likely be attributed to the presence of SHW, the observed health gains are likely due to both program components. Separate intervention trials are needed to assess the relative effectiveness of the health screenings and SHW programs. Third, the participation rates were somewhat higher among intervention schools than in controls. These differences seem to have resulted from slightly weaker recruitment at control schools, and could have caused selection bias if participating students were systematically different from students who did not join the study. The baseline data suggests that this was not the case, with nearly all covariates identical between intervention and control school students. Another limitation is the relatively small sample size and that the 14 schools in our sample were not randomly selected from a larger group of urban schools in Lusaka, and therefore does not allow us to draw general conclusions from the results presented. Lastly, the power of the study may be limited in detecting secondary outcomes since it was powered to detect a decline in infection prevalence.

This study contributes to the small body of evidence on the health of school-aged children in LMICs. The results presented here highlight the large unmet health needs of school-aged children and suggest that implementing a package of education, preventive, and clinical interventions through the school setting is feasible and confers large health benefits. Given its affordability and feasibility at scale, comprehensive school health programs appear to offer a highly effective platform for improving access to care and health status of school-age children.

## Supporting information

S1 Checklist TREND statement checklist. (PDF)

S1 Protocol Study protocol. (DOC)

S1 Table Mean of matched covariate by intervention status. (DOCX)

S2 Table Prevalence of acute illnesses. (DOCX)

S3 Table Cost of school health worker program and bi-annual health screenings. (DOCX)

S1 Appendix Acute illness questions. (DOCX)

S2 Appendix 11 question health knowledge quiz. (DOCX)

S1 Dataset Baseline and endline data. (DTA)

S1 Fig Proportion of study population not receiving treatment at baseline and endline. (TIF)
