# Supplementary material for: Comprehensive school-based health programs to improve child and adolescent health: Evidence from Zambia
Source: PLoS One. 2019 May 31;14(5):e0217893. doi: 10.1371/journal.pone.0217893 (PMC6544295; doi:10.1371/journal.pone.0217893)
Supplement: S1 Protocol — (DOC) [file pone.0217893.s002.doc]

**Instructions:** The purpose of this research protocol is to provide IRB members and reviewers with sufficient information to conduct a substantive review. If a separate sponsor’s protocol exists, please submit it in addition to this document.

Complete all of the sections below.  For more detailed instructions, please consult the Investigator’s Manual or IRB website (links provided below).

| **General Information** | |
| --- | --- |
| **Protocol #** (if assigned): | |
| **Version Date:** 21 April 2015 | **Version Number: Version # 1** |
| **Principal Investigator:** Günther Fink, PhD | |
| **Faculty Advisor (**if PI is a student**):** | |
| **Protocol Title:** Evaluating the Impact of School-based Health Programs on Children’s Well-being and Academic Performance | |

1. **Specific Aims**

The overall objective of the proposed research project is to determine whether children’s health and academic performance can be improved by a school-based health program currently piloted by a local NGO called *Angels of Mercy* in Lusaka, Zambia. The school health program has two components: the training of selected teachers to become “School Health Workers”, and bi-annual health screenings of all students.

In the proposed study, we hope to collect data on primary school students in Lusaka to address the following four specific aims:

1) Establish the prevalence of acute and chronic infections among primary school students in Lusaka.

2) Evaluate the impact of the school-based health programs (SBHP) on the prevalence of acute and chronic diseases among school going children.
3) Evaluate the impact of SBHP on school attendance and academic achievement.
4) Evaluate the impact of the School Health Worker training on teachers motivation and general school perceptions of school administrators, children and their mothers.

1. **Background**

**2.1 Provide the scientific background and rationale for the study**

While a lot of local and international efforts have focused on improving the health of children under the age of five in Zambia in recent years, relatively little is known regarding children’s wellbeing in late childhood. Two recent pilot studies suggest that children in this age group are suffering from a heavy exposure to both infectious and chronic health problems, which do not only undermine their physical development, but also limit their ability to attend school and excel as students. In this study, we propose to evaluate the effectiveness and impact of a new school-based health program (SBHP), which consists of two school-based interventions designed to improve children’s health:

1) *Bi-annual school health screenings*: Trained health workers (nurses trained by the government) come to schools every six months to i) provide children with vitamin A supplementation and deworming medication, ii) perform anthropometric measurements, and iii)test and examine children for the following conditions: schistosomiasis, acute respiratory infections, fungal infections, diarrheal diseases, malaria, tuberculosis, urinary tract infections, eye infections, and other acute and chronic conditions observed by the clinician during the physical examination.

2) A newly developed *School Health Worker program*: For the school health worker program, teachers are trained for 100 hours on the recognition of acute and chronic infections as well how to design and implement health education curricula, improve school water, sanitation and hygiene conditions (WASH), and provide basic first aid care such as management of trauma and bleeding, preparation of ORS, and pre-referral management. School administrators are trained for 45 hours on how to supervise, manage, and motivate school health workers, design school-wide health education curricula, and improve school WASH conditions.

*Background: Primary Schooling and Health Screenings*

According to the latest data from the World Bank and UNICEF, primary school enrollment in Zambia was 94% in 2012 and school attendance was over 81% between 2008 and 2012.^1,2^ The main sources of primary schooling in Zambia are government schools, private schools and community schools. Government schools are intended to be free for Zambians until grade 7, but they are not accessible by all: some charge fees for uniforms and other school supplies or are located in areas that make them inaccessible to some of the population.^3^ Community schools attempt to fill this gap by providing locally managed primary education to those in need. The quality of community schools, however, is inconsistent.^4^
School-based health screenings are a proven cost-effective method to manage preventable, treatable illnesses and improve school attendance in developing countries.^5-8^ Although the World Health Organization (WHO) recommends that all children in areas with high risk for helminth infection are treated with deworming medications, study results on the effectiveness of these interventions have been inconsistent. A study by Clarke et al. (2008) in western Kenya found that students in schools randomized to receive an intervention providing intermittent preventative treatment showed significant improvements in anemia, hemoglobin concentration, and sustained attention.^7^ Also in Kenya, Miguel and Kremer (2004) found that children in schools randomly assigned to a deworming intervention showed a one-quarter decrease in school absenteeism along with significant improvements in prevalence of hookworm, roundworm, schistosomiasis, and whipworm; self-reported health; and height for age.­^8^ A meta-analysis by Taylor-Robinson et al. (2012) found mixed results regarding the effects of deworming medications in children aged 16 and younger. Screening children for worms and then treating those who were infected appeared to increase weight and hemoglobin, but showed an unclear effect on cognitive functioning; single and multiple doses of deworming for all children without screening first, however, showed little effect on weight, hemoglobin, cognitive function, and school attendance. These results were not modified when stratified according to prevalence of helminth endemic in the area.^9^

*The new School Health Worker Program in Zambia*

In response to Zambia’s context and the health needs of young people, Healthy Kids/Brighter Future (HK/BF), an international NGO, in conjunction with Angels of Mercy (AoM), a registered Zambian NGO, and the Ng’ombe government health centre have recently developed a School Health Worker (SHW) program and piloted it in the Ng’ombe area. The SHW program is based on Zambia’s national Community Health Worker (CHW) model. The program’s purpose is to address issues of health system access and health education, with the broad goal of reducing the burden of preventable diseases that disproportionately affect children and young adults, including HIV/AIDS, diarrheal diseases, and nutritional deficiencies. The SHW program takes an innovative approach, working with school administration and training and empowering select teachers to conduct health lessons, perform first aid, and identify sick children that might require referral. The primary goal of this program is for SHWs to improve the health of young people through providing regular health education lessons and identifying negative health conditions in children in a timely manner and to ensure that the children receive prompt care. The SHW program also aims to empower the teachers who are trained to be SHWs.

To date, school-based health interventions in Zambia have focused mainly on health education.^10^ Between 2005-2009, USAID funded the Community Health and Nutrition, Gender and Education Program (CHANGES2) program supporting the Zambian Ministry of Education’s activities in four provinces, including Lusaka.^11^ CHANGES2 focused on strengthening teachers’ “professional skills,” as well as health education, particularly HIV/AIDS prevention. While it sought to expand schools’ health services, it did not employ teachers as health workers in schools.

**2.2 Describe the significance of the research, and how it will add to existing knowledge**

While free health care is provided for children under five years of age in Zambia, research on the health and well-being of the school-aged population in Zambia is limited. The newly designed SBHP currently piloted in 7 schools in Lusaka offer a potentially effective way to improve the health of students in Lusaka and elsewhere in the country; the aim of this project is to assess the effectiveness of these programs.

In our literature review, we could not identify any other program that utilizes a model similar to the SBHP program; for this reason among others, understanding if this innovative program is achieving its intended purpose is particularly important.

While improving population health through schools has long been a focus of the Zambian Ministry of Education, the SBHP model represents a new approach. School is an appropriate setting for a health intervention in Zambia because school-aged children are the population most affected by infectious diseases, and primary school attendance is as high as 94%.^1^ Additionally, no other known program utilizes a model similar to HK/BF’s SHW program in which teachers may make referrals to health centers. One program conducted by the Zambian Ministry of Education focused on strengthening teachers’ “professional skills” with in-service teacher trainings and follow-up “professional support”.^11^ While this program did work on developing leadership skills, it did not emphasize teacher empowerment, a point of the SHW program. Other school-based health interventions in Zambia focus mainly on health curriculum, such as the Nutrition Education in Basic Schools (NEBS) project also led by the Zambian Ministry of Education.^10^ Teachers in these other programs were also not trained to perform first aid or make referrals.

1. **Study Setting**
   1. **Identify the sites or locations where the research will be conducted.**

This study will be conducted in 14 primary schools in Lusaka district, Zambia.

- 1. **Describe the Principal Investigator’s experience conducting research at study site(s) and familiarity with local culture**The PI has extensive experience in Zambia. He has been conducting research there since 2009, and has established a close working relationship with partners at the University of Zambia as well as the Ministries of Education and Healthth.
  2. **Is the research conducted outside the United States?**

**No** **Yes: If yes; describe site-specific regulations or customs affecting the research, local scientific and ethical review structure**

The study will be conducted in Zambia and will need to be approved by a local IRB as well as the Ministry of Education – our application is currently under review.

- 1. **Are there any permissions that have been or will be obtained from cooperating institutions, community leaders, or individuals, including approval of an IRB or research ethics committee?** **No** **Yes: If yes; provide a list of the permissions (also include copies with the application, if available)**The research is under review by ERES Converge IRB in Lusaka, Zambia.

1. **Study Design**
   1. **Describe the study design type**

This study is designed as a prospective case-control study. Students from the seven intervention schools will be matched with students from control schools; all students will be assessed at baseline (prior to intervention rollout), midline (October) and endline (April 2016).

- 1. **Indicate the study’s duration - and the estimated date of study completion**

The study is projected to last one year. The estimated date of study completion will be June 2016. The proposed timeline is as follows:

| SHW training | April – May 2015 |
| --- | --- |
| Enrollment and baseline survey | May 2015 |
| Intervention rollout | Launch May/June 2015 |
| Midline survey | November 2015 |
| Endline survey & Qualitative analysis | April 2016 |
| Data analysis | May 2016 |
| Final report and data dissemination | June 2016 |

- 1. **Indicate the total number of participants (if applicable, distinguish between the number of participants who are expected to be screened and enrolled, and the number of enrolled participants needed)**

We expect to enroll approximately 1,100 students across 14 schools in this study.

- 1. **List inclusion criteria**

Children attending the 14 schools in the study.

- 1. **List exclusion criteria**

Children not attending any of the 14 target schools.

- 1. **Describe study procedures**

*Sampling*
Fourteen community schools will be involved in the study. In each school, three grades will be selected for the study; in treatment schools, two grades will be randomly selected among those directly taught by SHWs; the grades in the control group will be chosen to match the ones in the treatment group. To be able to link health status directly to standardized tests, all students in grade 7 will be invited to participate in the study in the 14 schools. Intervention schools will receive the SHW Program as well as baseline, midline, and endline health screenings. For each of the seven schools, we will identify a control school within Lusaka district. Control schools will be chosen based on the overall school size, number of pupils in each grade, and average test score performance.

*Outcome measures*
The primary outcome of the study will be child health (prevalence of acute and chronic infection at endline) as well as academic performance (school attendance and grade performance).

The quantitative assessments at baseline, midline and endline will be complemented by qualitative work. For the qualitative component, we will employ a non-probabilistic, stratified purposeful sampling methodology. In our study we will capture the opinions of SHWs, school administrators, mothers and children from a sample of community schools with the SHW program. We will use a mixture of qualitative research methods, namely semi-structured individual interviews and focus group discussions (FGDs).

We will sample a total of 10 SHWs across the seven intervention schools. Of the schools selected, we will randomly sample 3 schools for additional investigation. From this pool of 3 schools we will conduct individual interviews with the school administrators (preferably the headmaster), and FGDs with mothers and children. There will be a total of 3 administrator interviews (1 per school) and 7 FGDs with mothers and children (1 per intervention school).

*Internal validity*
In order to ensure that schools or students are not systematically different prior to the launch of the study (which could occur given the small sample sizes), classes and schools will be matched based on the overall school and class size, as well as the most recent school test performance. For each treated class, the most comparable class from the control group will be selected. A total of approximately 80 children (25-30 from each grade) will be enrolled from each school – all students in selected grades will be invited to join the study; final sample size will depend on class sizes as well as parental consent.

*Study interventions*
The study will assess (but not be directly involved with the delivery of) a combined intervention package currently implemented by a local NGO called Angels of Mercy. The program has two components:
1. The School Health Worker (SHW) Program. The aim of the SHW training is to empower teachers to recognize and refer children in need of medical attention, give basic first-aid care, and run health education lessons. Over the past year, HK/BF has worked with government officials and medical professionals in Zambia to develop a 10-day, 100-hour SHW training adapted from the Community Health Worker model. The curriculum trains teachers as front-line health workers capable of running health education lessons, recognizing and referring children in need of care, and providing first-line care.

Training sessions are facilitated by a group of health educators from the Ng’ombe Health Centre, all of whom have been certified by the Ministry of Health. The team of health educators consists of an environmental health-technician, clinician, clinic in-charge, and nutritionist. Additionally, all teachers are required to undergo twenty hours of clinical sessions at the Ng’ombe health centre, wherein they examine sick youth under the supervision of a clinical officer. Teachers receive K40 daily travel allowance (~$6) during training. The facilitation team from the Ng’ombe clinic meets with all of the SHWs at least once a month at their regular monthly meeting. During the meeting, the team is able to collect all forms from the teachers, assist the teachers to overcome challenges they have encountered, and add supplementary training lessons. The teachers are provided with a K100 monthly allowance (~$17), as well as shirts, bags, and SHW cards (which give them preferential care at the clinic).
Pre/post tests are conducted for all of the teachers in the SHW program. This test is re-administered at the ninety-day and half-year mark in order to measure how much information was retained by the teachers. Additionally, HK/BF and the Ng’ombe Health Centre collect a number of forms form the teachers and the health clinic in order to assess the performance of the teachers in their SHW roles. These include referral and feedback forms, examination registries, and health education reviews.
Selection of teachers for the training is done by the participant schools and the Ng’ombe Health Centre. The schools are able to nominate a pre-determined number of teachers to be interviewed by the in-charge and training facilitators from the Ng’ombe clinic. Following the interview, the clinic staff consults the school admin to select teachers to attend the training. The number of teachers trained from each school is dependent upon the number of pupils enrolled at the school. Schools with less than 200 pupils are able to send one teacher for training, schools with greater than 200 pupils send 2 teachers for training and schools with greater than 400 students are able to send 3 teachers. The target ratio for the program is at least 1 teacher for every 200 students.
School administrators will be trained for 45 hours on how to supervise, manage, and motivate school health workers, design school-wide health education curricula, and improve school WASH conditions. All school administrators receive a daily K50 travel allowance (~$8) for attending training and graduating administrators receive a t-shirt and certification.
2. Bi-annual school based preventive health screening days. In collaboration with the Ministry of Health bi-annual health screening days will be conducted at all target community schools. All health screenings will be conducted by nurses and clinical officers from the local government health clinic and supported by trained SHWs. Parents or guardians are expected to attend screening days with their children and informed consent is required by both the child and guardian. As part of the screening, a complete physical examination is performed including urine screening for schistosomiasis, which is endemic in the region. All children receive a dose of vitamin A, mebendazole for presumptive deworming, and, if indicated, Praziquantil for schistosomiasis. Medication for simple acute ailments will be dispensed to the guardian of sick pupils upon the completion of the health screening. All students with chronic or severe ailments will be referred to the local health centre. AoM offers health screenings to all students within a class and are not limited to the students whom will participate in the study.
*Control group:* 7 schools will be used as the control group. All control schools will complete a baseline survey including height/weight, mid upper-arm circumference, and self-reported health at baseline, as well as an endline survey including a complete health screening.

- 1. **Does the study involve the collection of data/specimens (including the use of existing data/specimens)?**

**No** **Yes: If yes; indicate how, when, where and from whom specimens or data will be obtained**

*Data collection instruments*
The study will use standard questionnaires for data collection. In addition to a full set of anthropometric measures (height, weight and mid-upper arm circumference) participants will be asked about their recent health history, health service access and treatment seeking. Students will also be asked to report on their own school performance. Teachers and school administrators will be asked to verify the scholastic performance of all students within the study.

*Midline and Endline assessment*Two rounds of follow-up assessments will be done: one in November 2015, and one in April 2016.

*Qualitative interviews and focus group discussions*
We intend to conduct semi-structured interviews with 10 SHWs in order to ask specific questions about the components of empowerment as established by the frameworks provided by the World Bank and CARE on measuring empowerment based on agency, structure and relations.^12,13^ We will sample one school administrator per school and conduct semi-structured interviews to elicit information about his or her perceptions of the SHWs' empowerment. These interviews can then be compared with those from the SHWs on their own sense of empowerment so we can better understand the difference between perceived and self-identified empowerment. To ensure that our interviews capture the concept of empowerment as it applies to this population, we will review the transcripts from the interviews as they are completed and make any necessary changes to protocol as the need arises.

Separately, we intend to conduct FGDs with mothers and children in order to investigate their perceptions of SHW empowerment. A focus group size of 8 will maximize the balance between too small, possibly limiting the chances of a lively and informative conversation, and too large, where that some voices might be drowned out and the conversation could veer off track.

- 1. **Is there a data and safety monitoring plan (required for greater than minimal risk studies)?**

**No** **Yes: If yes; describe the plan**

All identifiable data will be stored in a locked office in Lusaka; only de-identified data will be entered electronically and used for analysis.

- 1. **Are there any anticipated circumstances under which participants will be withdrawn from the research without their consent?**

**No** **Yes: If yes; describe the circumstances as well any associated procedures to ensure orderly termination**

1. **Data/Statistical Analyses Plan**
   1. **Briefly describe the plan for data analysis (including the statistical method if applicable)**

All outcome variables will be compared between treatment and control groups, controlling for baseline covariates observed. To control for within-school correlations, all standard errors will be clustered at the school level. Qualitative data will be analyzed using Grounded Theory, beginning with open coding, and following an iterative coding process.^13^ First, we will subject all data (SHW interviews, school administrator interviews, and both mother and student FGDs) to an open-coded process without prior assumption of theoretical frameworks. From this, key themes will be deduced and a codebook of these themes will be created. Codes will be generated and assessed using the qualitative analysis program NVivo.

- 1. **Is there a sample size/power calculation?**

**No** **Yes: If yes; describe the calculation and the scientific rationale, and, if applicable, by site and key characteristics such as participant demographics**

We expect a total sample size of approximately 1120 students across the 14 schools. The primary outcome will be pupil health, measured by the presence of any acute or chronic infection. The study is powered to detect a decline in infection prevalence from an expected baseline level of 0.50 to 0.35, assuming a design effect of 2 as well as an attrition rate of 10% over the study period. The secondary outcome of the study is academic performance; the primary measure we will use is days of school missed; the study is powered to detect a 20% increase in the proportion of students not missing any schooling.

1. **Recruitment Methods**
   1. **Does the study involve the recruitment of participants?**

**No: If no, skip to 7.1**

**Yes: If yes; indicate how, when, where, and by whom participants will be recruited**

All students in 3 selected grades at each school will be invited to participate in the study. Selected students will be asked to obtain their parents’ consent; students with completed consent forms will be enrolled in the study and asked to complete the baseline survey.

- 1. **Are there any materials that will be used to recruit participants, e.g., emails, posters, and scripts?**

**No** **Yes: If yes; provide a list of the materials (also include copies with the application)**

1. **Available Resources**
   1. **Describe the feasibility of recruiting the required number of participants within the recruitment period**

We do not anticipate any difficulties recruiting students. With an average of 25-30 students in each class we think that getting 75 students for each school should not be a problem.

- 1. **Describe how the Principal Investigator will ensure that a sufficient amount of time will be devoted to conducting and completing the research**

The project was designed to leave a sufficient amount of time to see impact; we have no specific deadline for finishing the research.

- 1. **Are there research staff members, in addition to the Principal Investigator?**

**No: If no, skip to** 7.5

**Yes: If yes; outline training plans to ensure that research staff members are adequately informed about the protocol and study-related duties**

The PI will work with Ms. Rachel Brigell, who will be the research coordinator for HSPH, and Mr. Lonnie Hackett, who will be the local coordinator. Both have completed research training and will work closely with the PI to make sure all protocols are followed.

- 1. **Describe the minimum qualifications for each research role (e.g., RN, social worker) their experience in conducting research, and their knowledge of local study sites and culture**

All school-based assessments will be conducted by nurses hired by HealthyKids/Brighter Future and trained by the study coordinator (Lonnie Hackett).

- 1. **Briefly describe how the research facilities and equipment at the research site(s) support the protocol’s aims, e.g., private rooms available for interviews, etc.**

The project will directly work with primary schools to make sure all interviews with students are conducted in a separate room or private space.

- 1. **Are there provisions for medical and/or psychological support resources (e.g., in the event of incidental findings, research-related stress)?**

**No** **Yes: If yes; describe the provisions and their availability**

1. **Vulnerable Populations**
   1. **Are there any potentially vulnerable populations (e.g., children, pregnant women, human fetuses, neonates, prisoners, elderly, economically disadvantaged, employees or students of the investigator or sponsor, undocumented, terminally ill, cognitively impaired or mentally ill, etc.)?**

**No: If no, skip to** 9.1

**Yes: If yes; identify all vulnerable populations**

We will be working with school children.

- 1. **Describe safeguards to protect their rights and welfare**

Child assent and parent consent will be obtained. Data will be stored securely as to avoid a breach of confidentiality. Students will be informed that participation is voluntary, and that they can exit the study (or not answer any question they find uncomfortable) at any time.

1. **Consent Process**
   1. **Will consent to participate be obtained?**

**No: If no, skip to** 9.5

**Yes: If yes; describe the setting, role of individuals involved, timeframe(s), and steps to minimize coercion/undue influence during the consent process (at the time of initial consent and throughout the study)**

We will work with school authorities to make sure all teachers and students are informed about the study. To minimize the risk of coercion, students will asked to assent and get their parents’ consent.

- 1. **Are there any special populations?**

**No** **Yes: If yes; describe the process to obtain consent, permission or assent**

- 1. **Will consent of the participants be documented in writing?**

**Yes** **No: If no; describe the rationale for requesting a waiver or alteration of documentation of consent (and/or parental permission)**

- 1. **Will participants be provided with a copy of their signed consent form or information sheet (when a consent form is not signed)?**

**Yes** **No: If no; explain any extenuating circumstances that make it impossible or inappropriate to meet this requirement, i.e., doing so may place participants at increased risk, if inadvertently disclosed**

- 1. **Is a waiver or alteration of consent (and/or parental permission) being requested?**

**No** **Yes: If yes; describe the rationale for the request. If the alteration is because of deception or incomplete disclosure, explain whether and how participants will be debriefed (include any debriefing materials with the application)**

Parental consent will be obtained because the study participants are children.

1. **Risks**
   1. **Are there any reasonably foreseeable risks, discomforts, and inconveniences to participants and/or groups/communities?**

**No** **Yes: If yes; indicate probability, magnitude, and duration of each (note that risks may be physical, psychological, social, legal, and/or economic)**

- 1. **Identify whether any of the information collected, if it were to be disclosed outside of the research, could reasonably place the participant at risk of criminal or civil liability or be damaging to the participant’s financial standing, employability or reputation.**

We do not think that any of the information collected about these children could put them in any kind of financial or reputational risk.

- 1. **Outline provisions in place to minimize risk**

We will ensure privacy for the interviews and make sure identifiable information will be stored safely in a locked office in Lusaka. Only de-identified data will be shared electronically and analyzied.

1. **Benefits**
   1. **Describe potential benefits of study participation (indicate if there is no direct benefit)**

Children will be screened for infections as part of the study endline, and will receive treatment if infections are found.

- 1. **Describe potential benefits of the research to the local community and/or society**

The results of this study may lead to improvements in health programs implemented in schools in Lusaka and elsewhere if the government decides to scale these programs.

1. **Reportable Events**
   1. **Outline plans for communicating reportable events (e.g., adverse events, unanticipated problems involving risks to participants or others, breach of confidentiality)**

Any adverse events will be directly reported to local IRB as well as to school administrators, who will reach out to parents as needed.

1. **Research Related Injuries (this section must be completed for any greater than minimal risk research)**

**13.1 Are there provisions for medical care and compensation for research-related injuries?**

**No** **Yes: If yes; outline these provisions (Please note that although Harvard’s policy is not to provide compensation for physical injuries that result from study participation, medical treatment should be available including first aid, emergency treatment and follow-up care as needed. If the research plan deviates from this policy, provide appropriate justification.)**

1. **Participant Privacy**
   1. **Describe provisions to protect participants’ privacy (their desire to control access of others to themselves, e.g., the use of a private interview room) and to minimize any sense of intrusiveness that may be caused by study questions or procedures**

All interviews will be conducted in a private room by trained study staff (nurses).

1. **Data Confidentiality**
   1. **Will the information that is obtained be recorded in such a manner that participants can be identified, directly or through identifiers linked to the participants?**

**No: If no, skip to 16.1**

**Yes: If yes; either state that participants will be told that their data will be public or describe provisions to maintain the confidentiality of identifiable data, e.g., use of password protections (please refer to the Harvard Research Data Security Policy Protection Memo, at** [**http://www.security.harvard.edu/harvard-research-data-security-policy-protection-memo**](http://www.security.harvard.edu/harvard-research-data-security-policy-protection-memo)**, for additional information about required data security measures) [NOTE: Harvard Research Data Security Policy does not always apply if data are not being stored at Harvard facilities. Please consult the Data Security Policy for additional information.]**

All identifiable data will be stored on paper files only, which will be stored in a locked office owned by HealthyKids/Brighter Future in Lusaka.

- 1. **Describe i) whether data will be transmitted, and if so how; ii) how long it will be stored; and iii) plans for the data at the end of the storage period (how will it be destroyed, or will it be returned to data provider)**

Only de-identified data will be submitted and analyzed.

- 1. **Indicate how research team members and/or other collaborators are permitted access to information about study participants**

Only the PI and his two co-investigators (Brigell & Hackett) will have access to the identifiable records (paper survey files). De-identified data may be shared with other researchers after publication of the final paper.

- 1. **If future open access, i.e., free availability and unrestricted use, of data is planned or likely, indicate how data will be released.**

De-identified data will be shared either through the journal webpage (if available) or through the Harvard Dataverse after publication.

1. **Costs and Payments**
   1. **Identify any costs that participants may incur during the study, including transportation costs, childcare, or other out-of-pocket expenses**

Participants will not incur any costs associated with the research.

- 1. **Is there any payment or reimbursement that participants may receive during the study?**

**No** **Yes: If yes; specify the amount, method and timing of disbursement. (Please refer to Harvard University Financial Policy on Human Subject Payments at** [**http://vpr.harvard.edu/sites/vpr.harvard.edu/files/news/Human%20Subject%20Payments%20Policy%20Final_0.pdf**](http://vpr.harvard.edu/sites/vpr.harvard.edu/files/news/Human Subject Payments Policy Final_0.pdf)**)**

1. **Multi-site Study Management**
   1. **Is this a multi-site study?**

**No** **Yes: If yes; describe plans for communication among sites regarding adverse events, interim results, protocol modifications, monitoring of data, etc.**

1. **Investigational Drug/Biologic/Device**
   1. **Does this study involve an Investigational Drug/Biologic/Device?**

**No: If no; skip to** 19.1

**Yes: If yes; identify and describe the drug/biologic/device (e.g., marketing status: Is there an IND/IDE, classification of a device as significant vs. non-significant risk)**

- 1. **Describe its administration or use**
  2. **Compare the research drug/biologic/device to the local standard of care**
  3. **Describe plans for receiving, storage, dispensing and return (to ensure that they will be used only for participants and only by authorized investigators)**
  4. **If proven beneficial, describe anticipated availability and cost to participants post-study; plans (if applicable) to make available**

1. **HIPAA Privacy Protections**
   1. **Are HIPAA privacy protections required? Please note that only Harvard University Health Services and Harvard School of Dental Medicine are covered entities at Harvard. Harvard is otherwise not a HIPAA covered entity. If, however, data is derived from a Covered Entity (e.g. a hospital or community health center), mark ‘yes’ and address the items below.**

**No: If no; skip to** 20.1

**Yes: If yes; include at least one of the following:**

**Describe plans for obtaining authorization to access protected health information**

**Provide the rationale for a waiver of authorization or limited waiver of authorization request**

1. **Data and Specimen Banking**
   1. **Does the study include Data and Specimen Banking?**

**No: If no; skip to** 21.1

**Yes: If yes; identify what will be collected and stored, and what information will be associated with the specimens**

- 1. **Describe where and how long the data/specimens will be stored and whether participants’ permission will be obtained to use the data/specimens in other future research projects**
  2. **Identify who may access data/specimens and how**
  3. **Will specimens and/or data be sent to research collaborators outside of Harvard?**

**No** **Yes: If yes; describe the plan**

- 1. **Will specimens and/or data be received from collaborators outside of Harvard?**

**No** **Yes: If yes; describe the plan**

1. **Sharing Study Results**
   1. **Is there a plan to share study results with individual participants?**

**No** **Yes: If yes; describe the plan**

- 1. **Is there a plan to disseminate aggregate results to the community where the research is conducted?**

**No** **Yes: If yes; describe the plan**

We will hold a dissemination meeting in Lusaka at the end of the study (end of 2016) to share results with school boards as well as the Ministries of Health and Education.

1. **Regulatory Compliance**
   1. **Describe plan for monitoring regulatory compliance, in order to ensure proper record keeping and retention of required regulatory documents**

The PI will be in weekly contact with the study coordinator to make sure all protocols are followed. He will also visit Lusaka in July to personally inspect the site and ensure protocols are followed. Ms. Brigell will be in Lusaka from May to July to actively work on the project.

References

1. World Bank. (2014). School enrollment, primary (% net). Retrieved from http://data.worldbank.org/indicator/SE.PRM.NENR.

2. UNICEF. (2013). Zambia: Statistics. Retrieved from: http://www.unicef.org/infobycountry/zambia_statistics.html.

3. Agnew-Blais, J., Carnevale, J., Gropper, A., Shilika, E., Nyimbili, E., Mukuka, S., & Mitchell, D. (2007). Report on the Health of Community School Children in Ng’ombe Compound, Lusaka, Zambia (June - August 2007). Unpublished manuscript, Harvard School of Public Health, Boston, MA, Harvard Medical School, Boston, MA, Stanford University, Stanford, CA, & University of Zambia School of Medicine, Lusaka, Zambia.

4. UNICEF. (2001). ZAM: Educating Children Out of the System: The Community Schools Movement in Zambia. Retrieved from: http://www.unicef.org/evaldatabase/index_31221.html.

5. Jukes, M.C.H., Drake, L.J., & Bundy, D.A.P. (2008). School Health, Nutrition and Education for All: Levelling the Playing Field. CABI Publishing, Wallingford, UK.

6. Fernando, D., de Silva, D., Carter, R., Mendis, K.N., & Wickremasin, R. (2006). A randomized, double-blind, placebo-controlled, clinical trial of the impact of malaria prevention on the educational attainment of schoolchildren. *American Journal of Tropical Medicine and Hygiene, 74*, 386–393.

7. Clarke, S.E., Jukes, M.C.H., Njagi, J.K., Khasakala, L., Cundill, B., Otido, J., Crudder, C., Estambale, B.B.A., & Brooker, S. (2008). Effect of Intermittent Preventive Treatment of Malaria on Health and Education in Schoolchildren: a Cluster- randomised, Double-blind, Placebo-controlled Trial. *Lancet*, *372*:127-38.

8. Kremer, M., &Miguel, E. (2004). Worms: Identifying Impacts on Education and Health in the Presence of Treatment Externalities *Econometrica, 72*(1):159 - 217.

9. Taylor-Robinson, D.C., Maayan, N., Soares-Weiser, K., Donegan, S., & Garner, P. (2012). Deworming drugs for soil-transmitted intestinal worms in children: effects on nutritional indicators, haemoglobin and school performance. *The Cochrane Library, 11*.

10. Sherman, J. and Muehlhoff, E. (2007). Developing a nutrition and health education program for primary schools in Zambia. Journal of Nutrition Education and Behavior, 39(6): 355-342.

11. American Institutes for Research (2009). Zambia’s community health and nutrition, gender and education support 2 program (CHANGES2). Retrieved from http://www.air.org/project/zambia%E2%80%99s-community-health-and- nutrition-gender-and-education-support-2-program-changes2.

12. Alsop, R. and Heinsohn, N. (2005). Measuring empowerment in practice: structuring analysis and framing indicators (Working Paper 3510). Retrieved from World Bank Policy Research website: <http://siteresources.worldbank.org/INTEMPOWERMENT/Resources/41307_wps 3510.pdf>.

13. CARE (n.d.). *Women Empowerment SII Framework.* Retrieved from http://pqdl.care.org/sii/Pages/Women%27s%20Empowerment%20SII%20Frame.

14. Strauss, A., & Corbin, J. (1998). Basics of qualitative research: Techniques and procedures for developing grounded theory (2nd ed.). Thousand Oaks, CA: Sage.
