# Supplementary material for: Comprehensive school-based health programs to improve child and adolescent health: Evidence from Zambia
Source: PLoS One. 2019 May 31;14(5):e0217893. doi: 10.1371/journal.pone.0217893 (PMC6544295; doi:10.1371/journal.pone.0217893)
Supplement: S1 Table — (DOCX) [file pone.0217893.s003.docx]

| **Matched Covariate** | **Intervention** | **Control** | **P-value** |
| --- | --- | --- | --- |
| Number of Students | 304.29 | 377 | 0.43 |
| Number of Teachers | 10.71 | 10.71 | 1 |
| Passing Rate | 0.879 | 0.846 | 0.69 |
