# Supplementary material for: Comprehensive school-based health programs to improve child and adolescent health: Evidence from Zambia
Source: PLoS One. 2019 May 31;14(5):e0217893. doi: 10.1371/journal.pone.0217893 (PMC6544295; doi:10.1371/journal.pone.0217893)
Supplement: S2 Table — (DOCX) [file pone.0217893.s004.docx]

|  |  | **BASELINE** | | **ENDLINE** | |
| --- | --- | --- | --- | --- | --- |
| **Illness Outcome** | **Group** | **%** | **95% CI** | **%** | **95% CI** |
| *Diarrhea* | Intervention | 21.47 | (16.37- 29.01) | 12.15 | (7.82- 17.92) |
|  | Control | 24.47 | (18.05- 30.90) | 18.28 | (13.0- 24.6) |
| *Fever* | Intervention | 32.63 | (26.65- 40.94) | 12.22 | (7.87- 18.01) |
|  | Control | 36.9 | (29.28-43.70) | 21.2 | (15.53- 27.82) |
| *Itchy Eyes* | Intervention | 13.61 | (9.09-19.31) | 3.31 | (1.24-7.11) |
|  | Control | 7.41 | (4.11-12.12) | 4.32 | (1.89-8.34) |
| *Itchy eyes with eye pus* | Intervention | 6.81 | (3.67-11.36) | 5.52 | (2.7-9.97) |
|  | Control | 11.64 | (7.44-17.09) | 8.65 | (5.02-13.66) |
| *Cough with fast breathing* | Intervention | 25 | (18.86-31.99) | 6.67 | (3.5-11.36) |
|  | Control | 10.33 | (6.33-15.66) | 11.29 | (7.13-16.73) |
| *Cough with chest pain* | Intervention | 35 | (28.05-42.45) | 21.11 | (15.39-27.8) |
|  | Control | 48.9 | (41.49-56.73) | 30.65 | (24.11-37.81) |
| *Cough with thick fluid* | Intervention | 23.33 | (17.37-30.20) | 9.44 | (5.6-14.69) |
|  | Control | 27.7 | (21.39-34.78) | 30.64 | (24.1-37.81) |
| *Skin rash* | Intervention | 17.8 | (12.49- 24.16) | 8.84 | (5.17- 14.03) |
|  | Control | 23.81 | (16.97- 29.58) | 8.11 | (4.61- 13.02) |
| *Blood in urine* | Intervention | 4.71 | (2.18-8.76) | 1.11 | (0.14-3.98) |
|  | Control | 3.72 | (1.51-7.52) | 3.76 | (1.53-7.6) |
| *Painful urination* | Intervention | 12.63 | (8.26- 18.21) | 7.18 | (3.90- 12.03) |
|  | Control | 16.93 | (11.89 -23.05) | 3.76 | (1.53- 7.60) |
| *Frequent night urination* | Intervention | 11.35 | (7.17-16.83) | 5.52 | (2.7-9.98) |
|  | Control | 13.37 | (8.84-19.1) | 9.94 | (6.0-15.26) |
| *Red or brown urine* | Intervention | 10.44 | (6.36- 16.13) | 11.6 | (7.37- 17.28) |
|  | Control | 8.74 | (4.79- 13.52) | 14.05 | (9.39-19.91) |
| *Mucus in stool* | Intervention | 6.42 | (3.36-10.94) | 2.78 | (0.91-6.4) |
|  | Control | 6.35 | (3.32-10.82) | 0.54 | (0.014-2.97) |
| *Worms in stool* | Intervention | 15.26 | (10.47-21.18) | 2.76 | (0.91-6.36) |
|  | Control | 32.09 | (25.46-39.29) | 7.53 | (4.18-12.31) |
