# Supplementary material for: Comprehensive school-based health programs to improve child and adolescent health: Evidence from Zambia
Source: PLoS One. 2019 May 31;14(5):e0217893. doi: 10.1371/journal.pone.0217893 (PMC6544295; doi:10.1371/journal.pone.0217893)
Supplement: S3 Table — (DOCX) [file pone.0217893.s005.docx]

| **ENROLLMENT AT STUDY INTERVENTION SCHOOLS** | | |  |
| --- | --- | --- | --- |
| Average school enrollment | 352 |  |  |
| Average student/SHW (direct) | 100 |  | Size of class taught by SHW |
| Average student/SHW (indirect) | 176 |  | Total school enrollment/#SHWs |
|  |  |  |  |
| **COST BREAKDOWN** | **Year 1 (Implementation)** | **Year 2 (Ongoing)** |  |
| **A. School Health Worker Program** |  |  |  |
| Training | $210.00 | $0.00 |  |
| Initial equipment | $65.00 | $0.00 |  |
| Ongoing support | $165.00 | $165.00 |  |
| **TOTAL** | **$440.00** | **$165.00** |  |
|  |  |  |  |
| Average cost per student (direct) | $4.40 | $1.65 | Average cost per child taught by SHW |
| Average cost per student (indirect) | $2.50 | $0.94 | Average cost per child taught at school |
|  |  |  |  |
| **B. Biannual Health Screenings*** |  |  |  |
| Average cost per school | $245.00 | $160.00 |  |
| Average cost per student | $0.70 | $0.45 |  |
|  |  |  |  |
| **First screening includes physical assessment, anthropometric measurements, deworming and vitamin A. Second screening includes all except physical assessment. Full Screening = $0.47, Deworming & Vitamin A only = $0.23* | | | |
| **TOTAL COST (PROGRAMS A + B)** | **Year 1 (Implementation)** | **Year 2 (Ongoing)** |  |
| Average cost per student (direct) | $5.10 | $2.10 | Average cost per child taught by SHW |
| Average cost per student (indirect) | $3.20 | $1.39 | Average cost per child taught at school |
