# Supplementary material for: Comprehensive school-based health programs to improve child and adolescent health: Evidence from Zambia
Source: PLoS One. 2019 May 31;14(5):e0217893. doi: 10.1371/journal.pone.0217893 (PMC6544295; doi:10.1371/journal.pone.0217893)
Supplement: S1 Appendix — (DOCX) [file pone.0217893.s006.docx]

**4. Systemic**

4-1) In the last 2 weeks, have you had a fever? **(Please circle)**

1. Yes 2. No 3. Don’t know 4. Refuse

4-4) In the last 2 weeks, have you had night sweats? **(Please circle)**

1. Yes 2. No 3. Don’t know 4. Refuse

**5. Gastrointestinal**

5-1) In the last 2 weeks, have you had diarrhea? **(Please circle)**

1. Yes 2. No 3. Don’t know 4. Refuse

5-4) In the last 2 weeks, have you had any blood in the stool? **(Please circle)**

1. Yes 2. No 3. Don’t know 4. Refuse

5-5) Mucus is a slippery, whitish substance: In the last 2-weeks, have you had any mucus in the stool? **(Please circle)**

1. Yes 2. No 3. Don’t know 4. Refuse

5-6) Have you ever seen any worms in your vomit or stool? **(Please circle)**

1. Yes 2. No 3. Don’t know 4. Refuse

**6. Respiratory**

6-1) In the last 2 weeks, have you had an illness with cough? **(Please circle)**

1. Yes 2. No 3. Don’t know 4. Refuse

**If YES TO 6-1:** 6-2) Did you have chest pain at the same time as the cough was present? **(Please circle)**

1. Yes 2. No 3. Don’t know 4. Refuse

**If YES TO 6-1:** 6-3) Did you cough up any thick fluid that you could hear? **(Please circle)**

1. Yes 2. No 3. Don’t know 4. Refuse

**If YES TO 6-1:** 6-4) When you had an illness with a cough, did you have difficult or fast breathing? **(Please circle)**

1. Yes 2. No 3. Don’t know 4. Refuse

**If YES to 6-4:** 6-5) When you had an illness with a cough and difficult and/or fast breathing, did you also have body hotness? **(Please circle)**

1. Yes 2. No 3. Don’t know 4. Refuse

**If YES to 6-4**: 6-6) Did you seek advice or treatment for the cough/fast breathing? **(Please circle)**
 1. Yes 2. No 3. Don’t know 4. Refuse

**7. Genitourinary**

7-1) What color is your urine? **(Please circle one)**

1. Yellow
2. Clear
3. Brown
4. Red
5. Other (please specify):_____________________
6. Don’t know
7. Refuse

7-2) In the past 2 weeks, have you had any blood in the urine? **(Please circle)**

1. Yes 2. No 3. Don’t know 4. Refuse

7-3) In the past two weeks, have you had any pain when urinating? **(Please circle)**

1. Yes 2. No 3. Don’t know 4. Refuse

7-4) In the past two weeks, did you have to get up more than 3 times in the night to urinate? **(Please circle)**

1. Yes 2. No 3. Don’t know 4. Refuse

**8. Skin, Vision, Thyroid**

8-1) In the last 2 weeks, have you had any visible pimply rash? **(Please circle)**

1. Yes 2. No 3. Don’t know 4. Refuse

8-3) In the last 2 weeks, have you had watery and itchy red eyes? **(Please circle)**

1. Yes 2. No 3. Don’t know 4. Refuse

**If YES to 8-3:** 8-4) Were your red, itchy eyes accompanied with pus or discharge? **(Please circle)**

1. Yes 2. No 3. Don’t know 4. Refuse

**Supplementary Appendix 2. 11 Question health knowledge quiz**

**10. Knowledge on Health Topics**

10-1) Have you ever heard of malaria? **(Please circle)**

1. Yes 2. No 3. Don’t know 4. Refuse

10-2) What is the most effective method for preventing malaria? **(Please circle)**

1. Bed nets

2. Spraying

3. Lotions

4. Other

5. Don’t know

6. Refuse

10-3) Have you ever heard of HIV/AIDS? **(Please circle)**

1. Yes 2. No 3. Don’t know 4. Refuse

10-4) Have you ever been tested for HIV? **(Please circle)**

1. Yes 2. No 3. Don’t know 4. Refuse

10-5) Which of the following is a way that a person can get HIV/AIDS? **(Read options and then circle)**

1. Mosquitos

2. Sharing sharp needles

3. Witchcraft

4. Kissing

5. Don’t know

6. Refuse

10-6) If water is clear does that mean it is safe to drink? **(Please circle)**

1. Yes 2. No 3. Don’t know 4. Refuse

10-7) Have you ever heard of tuberculosis (TB)? **(Please circle)**

1. Yes 2. No 3. Don’t know 4. Refuse

10-8) How can a person get tuberculosis? **(Please read options and circle all that apply)**

1. Mosquitos

2. Being near someone who is infected

3. Witchcraft

4. Sharing sharp needles

5. Don’t know

6. Refuse

10-9) Is it okay to stop taking your TB medicine early if you begin to feel better? **(Please circle)**

1. Yes 2. No 3. Don’t know 4. Refuse

10-10) Have you ever heard of bilharzia? **(Please circle)**

1. Yes 2. No 3. Don’t know 4. Refuse

10-11) What is the most common sign that someone has bilharzia? **(Please circle)**

1. Diarrhea

2. Blood in urine

3. Fever

4. Coughing

5. Don’t know

6. Refuse

10-12) How can someone get bilharzia? **(Please circle)**

1. Eating uncooked food

2. Kissing someone with bilharzia

3. Swimming in infected water

4. Mosquitos

5. Don’t know

6. Refuse
