# Supplementary material for: Comprehensive school-based health programs to improve child and adolescent health: Evidence from Zambia
Source: PLoS One. 2019 May 31;14(5):e0217893. doi: 10.1371/journal.pone.0217893 (PMC6544295; doi:10.1371/journal.pone.0217893)
Supplement: S2 Appendix — (DOCX) [file pone.0217893.s007.docx]

**10. Knowledge on Health Topics**

10-1) Have you ever heard of malaria? **(Please circle)**

1. Yes 2. No 3. Don’t know 4. Refuse

10-2) What is the most effective method for preventing malaria? **(Please circle)**

1. Bed nets

2. Spraying

3. Lotions

4. Other

5. Don’t know

6. Refuse

10-3) Have you ever heard of HIV/AIDS? **(Please circle)**

1. Yes 2. No 3. Don’t know 4. Refuse

10-4) Have you ever been tested for HIV? **(Please circle)**

1. Yes 2. No 3. Don’t know 4. Refuse

10-5) Which of the following is a way that a person can get HIV/AIDS? **(Read options and then circle)**

1. Mosquitos

2. Sharing sharp needles

3. Witchcraft

4. Kissing

5. Don’t know

6. Refuse

10-6) If water is clear does that mean it is safe to drink? **(Please circle)**

1. Yes 2. No 3. Don’t know 4. Refuse

10-7) Have you ever heard of tuberculosis (TB)? **(Please circle)**

1. Yes 2. No 3. Don’t know 4. Refuse

10-8) How can a person get tuberculosis? **(Please read options and circle all that apply)**

1. Mosquitos

2. Being near someone who is infected

3. Witchcraft

4. Sharing sharp needles

5. Don’t know

6. Refuse

10-9) Is it okay to stop taking your TB medicine early if you begin to feel better? **(Please circle)**

1. Yes 2. No 3. Don’t know 4. Refuse

10-10) Have you ever heard of bilharzia? **(Please circle)**

1. Yes 2. No 3. Don’t know 4. Refuse

10-11) What is the most common sign that someone has bilharzia? **(Please circle)**

1. Diarrhea

2. Blood in urine

3. Fever

4. Coughing

5. Don’t know

6. Refuse

10-12) How can someone get bilharzia? **(Please circle)**

1. Eating uncooked food

2. Kissing someone with bilharzia

3. Swimming in infected water

4. Mosquitos

5. Don’t know

6. Refuse
