# Supplementary figures and images for: Comprehensive school-based health programs to improve child and adolescent health: Evidence from Zambia
Source: PLoS One. 2019 May 31;14(5):e0217893. doi: 10.1371/journal.pone.0217893 (PMC6544295; doi:10.1371/journal.pone.0217893)

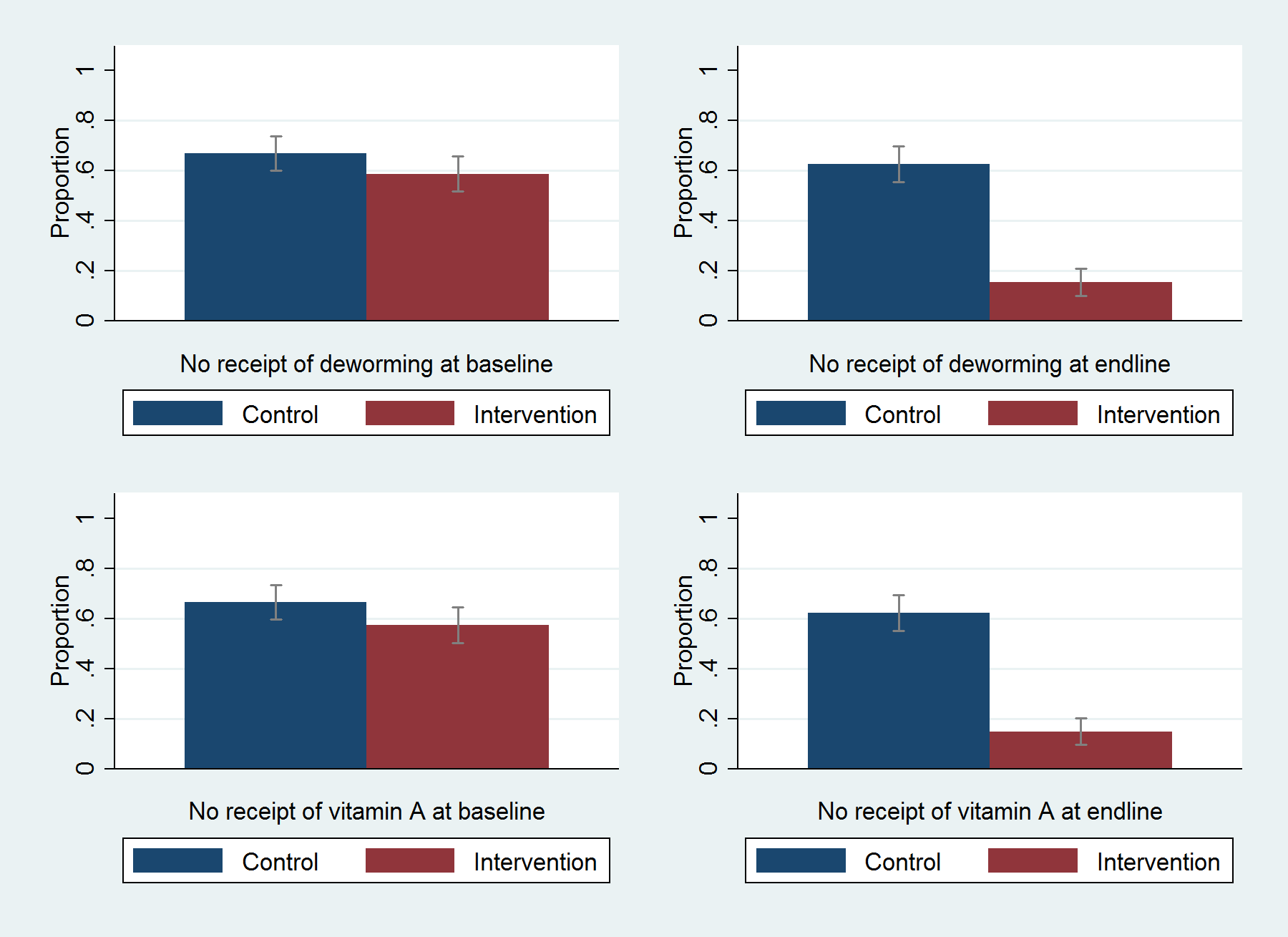

Supplement: S1 Fig — (TIF) [file pone.0217893.s009.tif]
